# First Incursion of *Salmonella* *enterica* Serotype Typhimurium DT160 into New Zealand

**DOI:** 10.3201/eid0904.020439

**Published:** 2003-04

**Authors:** Craig N. Thornley, Greg C. Simmons, Megan L. Callaghan, Carolyn M. Nicol, Michael G. Baker, Kylie S. Gilmore, Nicholas K.G. Garrett

**Affiliations:** * Institute of Environmental Science & Research Limited, Kenepuru Science Centre, Porirua, New Zealand; † Auckland Regional Public Health Service, Auckland District Health Board, Auckland, New Zealand; ‡ Mt. Albert Science Centre, Auckland, New Zealand

**Keywords:** Disease outbreaks, Salmonella infections/epidemiology, Salmonella typhimurium, case-control studies, zoonoses, birds, New Zealand, dispatch

## Abstract

An outbreak of human *Salmonella* *enterica* serotype Typhimurium DT160 infection in New Zealand was investigated from May to August 2001. Handling of dead wild birds, contact with persons with diarrheal illness, and consumption of fast food were associated with infection. Contaminated roof-collected rainwater was also detected.

Although rates of reported salmonellosis in New Zealand are relatively high for an industrialized country (average of 46.1 cases per 100,000 population per year, 1995–2001 [*1*]), strict biosecurity has prevented extension to New Zealand of the global pandemics of *Salmonella enterica* serotype Enteritidis and *S. enterica* serotype Typhimurium definitive type (DT) 104 (*2*). *S*. Typhimurium DT160 was first identified as a human pathogen in New Zealand in 1998 (*3*). Since July 2000, the incidence of human infection with this serotype has increased markedly, and the geographic distribution of cases has progressively expanded from New Zealand’s South to North Islands (Baker et al., unpub. data). However, routes of disease transmission have not been identified. The epidemic occurred in parallel with an epizootic leading to deaths in wild birds, mainly sparrows, due to septicemia caused by *S.* Typhimurium DT160 (*4*). We report an investigation of an outbreak of *S.* Typhimurium DT160 infection in humans.

## The Study

In May 2001, 24 cases of *S.* Typhimurium DT160 salmonellosis were reported in the Auckland region compared with an average of four sporadic cases each month with this serotype in the previous 7 months. Raw and undercooked egg consumption was commonly reported by the first 10 case-patients interviewed. A case-control study and environmental investigation were undertaken to identify the vehicle of infection and source of the outbreak. Recognizing the potential for a widely dispersed foodborne outbreak, we expanded the investigation throughout New Zealand.

Cases were identified from disease reports and isolates received by the national reference laboratory. We defined a case as diarrhea (≥3 loose stools in a 24-hour period) or vomiting after April 28, 2001, with a stool specimen positive for *S.* Typhimurium DT160. Patients were excluded if they had a history of contact with another person with culture-confirmed *S.* Typhimurium DT160 infection, or if they had a history of recent overseas travel. Each case was matched with two controls found from randomly drawn telephone numbers, matching for neighborhood and age (<1, 1–4, 5–14, >14 years).

Patients and controls were interviewed by telephone. The questionnaire covered symptoms (patients only) and contact with other symptomatic persons, bird or animal contact, and food consumption in the 3-day period before onset of illness (cases and controls). Parents or guardians were interviewed on behalf of children ages <12 years. A matched univariate analysis was performed with SAS software (*5*). Stepwise conditional logistic regression analyses were performed, also using SAS, to identify the combination of variables that best explained the differences between case-participants and controls.

Samples from the drinking water supply of case-patients with a history of recent consumption of nonreticulated water were collected and tested for coliforms and *S. enterica* by using standard methods (*6*). Brands of eggs eaten raw within the incubation period were sampled at random from retail displays at the case-patients’ purchase site. At least six shell eggs were collected in each sample. Eggshell surfaces and contents were tested with standard methods (*7*). Broken or cracked eggs were excluded from analysis. *Salmonella* isolates were serotyped by using the Kauffman-White scheme (*8*) and *S.* Typhimurium isolates were phage typed by using the Laboratory of Enteric Pathogens method (*9*).

From May to August 2001, a total of 170 case-patients meeting the case definition were identified. Of these, 119 (70%) agreed to participate and were enrolled in the study, along with 235 matched controls. The median age of case-patients was 8 years (range 4 months to 90 years), and 57% were female. The most frequently reported symptoms were diarrhea (97%), abdominal pain (77%), excessive tiredness (67%), and fever (66%). Vomiting in the absence of diarrhea was reported by one (0.8%) patient. The median duration of illness was 7 days (range 1–44 days); 17 (15%) patients were hospitalized, and none died. Case-patients and controls did not differ significantly according to age, sex, immunosuppressive therapy, treatment to reduce gastric acidity, or use of antibiotics. All *S.* Typhimurium DT160 isolates were sensitive to ampicillin, cephalothin, chloramphenicol, ciprofloxacin, co-trimoxazole, gentamicin, streptomycin, sulfonamides, tetracycline, and trimethoprim.

Seven exposures had significant univariate associations with increased risk for illness (Table). Four represented different levels of contact with other persons with gastrointestinal illness (i.e., within 28 days of illness onset; within 3 days of onset; within the household; or outside the household). Direct handling of dead wild birds, consumption of fast food, and consumption of food at a large gathering, such as at a party or large barbecue, were also significantly associated with illness. Six of those who had handled dead birds were <5 years of age. Others who had handled dead birds had no characteristics in common. After stepwise regression, contact with a person with gastrointestinal illness in the 28 days before onset of illness in the case-patient (adjusted odds ratio [OR] 2.8; 95% confidence interval [CI] 1.4 to 5.4), exposure to dead wild birds (adjusted OR 10.5; 95% CI 2.3 to 47.5), and consumption of fast food (adjusted OR 1.7; 95% CI 1.0 to 2.9) had independent significant associations with illness.

**Table:** Frequency of selected exposures among case-patients and controls, *Salmonella* Typhimurium DT160 outbreak, New Zealand, 2001^a^

Exposure	No. (%)		Matched OR	95% CI	p value
	Case-patients n = 119	Controls n = 235			
Direct handling of a dead wild birds	13 (10.9)	3 (1.3)	12.28	2.76 to 54.63	0.001
Exposure to person with D&V in household in 3 d before illness in case-patient	7 (5.9)	3 (1.3)	4.67	1.21 to 18.05	0.03
Exposure to person with D&V in any setting in 3 d before illness in case-patient	14 (11.8)	8 (3.4)	3.81	1.53 to 9.49	0.004
Exposure to person with D&V in household in 28 d before illness in case-patient	11 (9.2)	8 (3.4)	3.11	1.13 to 8.54	0.03
Exposure to person with D&V in any setting in 28 d before illness in case-patient	28 (23.5)	20 (8.5)	3.05	1.64 to 5.69	<0.001
Consumption of food at a large gathering	24 (20.2)	23 (9.8)	2.44	1.27 to 4.68	0.007
Consumption of any fast food	69 (58.0)	111 (47.2)	1.69	1.04 to 2.75	0.04
Drinking of roof-collected rainwater	12 (10.1)	19 (8.1)	2.35	0.55 to 10.05	0.25
Consumption of raw eggs	5 (4.2)	3 (1.3)	3.33	0.80 to 13.95	0.10

^a^DT, definitive type; D&V, diarrhea and vomiting; OR, odds ratio; CI, confidence interval.

Twelve case-patients throughout New Zealand indicated that they had drunk water from nonreticulated and untreated water sources. Eight sources were sampled in Auckland. Seven patients used roof-collected rainwater, and one used rainwater plus water from a dam. Four sampled sources, used by five patients, contained *S.* Typhimurium DT160. All were from roof-collected rainwater. Four of the five patients who had eaten raw eggs could identify the retail brand and outlet of purchase. These four patients had purchased six different brands of eggs from seven different retail outlets. Samples for two brands were positive for *S.* Thompson, both from shell surface washings.

## Conclusions

Epidemiologic investigation of an outbreak of *S.* Typhimurium DT160 infection in New from May to August 2001 found that contact with dead wild birds, contact with other persons with gastrointestinal illness, and consumption of fast food were all significantly associated with illness. In addition, *S.* Typhimurium DT160 was found in roof-collected rainwater drunk by five patients.

*S.* Typhimurium DT160 had been previously identified as the cause of large numbers of sparrow deaths in New Zealand in 2000, and analysis by pulsed-field gel electrophoresis (using the method described by Barrett et al. [*10*] and restriction enzyme *Xba*I) demonstrated that bird and human isolates in 2000 were indistinguishable (*4*). In our study, information was not collected on exposure to environments contaminated by wild bird feces, such as parks and play areas, a fact that may have underestimated the avian contribution to human illness. *S.* Typhimurium DT160 has previously been recognized as a bird pathogen in Canada (*11*) and in England (*12*). Before its emergence in New Zealand, the human *S.* Typhimurium DT160 infection had only been reported in the context of a 1979 institutional outbreak in the United Kingdom, linked to food contamination by sparrow droppings (*13*).

Consumption of undisinfected water has previously been identified as a risk factor for salmonellosis linked to bird transmission (*14*). This risk factor was not confirmed by our case-control study, despite the finding of *S.* Typhimurium DT160 in roof-collected rainwater. This discrepancy is probably because case-patients and controls were matched by neighborhood, and types of water sources are usually consistent within neighborhoods.

The association of illness with contact with another person with gastrointestinal illness is likely underestimated because secondary salmonellosis cases were excluded. Consumption of fast food was associated with illness; however, no single type of food outlet or food was identified. Case-patients were equally likely to have eaten food from chain fast-food restaurants as from family-owned fast-food outlets. Consumption of fast food may have occurred in environments contaminated by bird feces, or the foods themselves may have been contaminated, either during production or by infected foodhandlers (*15*).

Sampling and recall bias may have influenced the results of this study. Asymptomatic *Salmonella* carriers would not have been excluded from selection as controls, potentially reducing the magnitude of observed associations. Recall may have been influenced by delays between exposure and interview, although participants were asked to refer to a memory aid (personal diary or calendar). Recall of unusual exposures is less likely to have been affected.

The investigation successfully excluded a single common source exposure for this outbreak and instead suggested that multiple exposures contribute to *S.* Typhimurium DT160 infections in New Zealand. Strategies for addressing these exposures include routine treatment of roof-collected rainwater, hygienic disposal of dead birds, and promotion of hand-hygiene protocols and sick foodhandler policies in fast-food outlets. The source of this incursion of *S.* Typhimurium DT160 into New Zealand remains unknown: Bird isolates have been exclusively from nonmigratory birds, *S.* Typhimurium DT160 has not been identified in neighboring countries in the Pacific Basin, and early case-patients did not have a history of overseas travel.

## References

[1] Thornley C, Baker M, Nicol C. The rising incidence of salmonellosis in New Zealand, 1995–2001. New Zealand Public Health Report. 2002;9:25–8.

[2] Crump JA, Murdoch DR, Baker MG. Emerging infectious diseases in an island ecosystem: the New Zealand perspective. Emerg Infect Dis. 2001;7:767–72. 10.3201/eid0705.010501 11747690 PMC2631882

[3] Callaghan M, Simmons G. Outbreak of *Salmonella* Typhimurium phage type 160 in Auckland linked to an umu function. New Zealand Public Health Report. 2001;8:44–5.

[4] Alley MR, Connolly JH, Fenwick SG, Mackereth GF, Leyland MJ, Rogers LE, An epidemic of salmonellosis caused by *Salmonella* Typhimurium DT160 in wild birds and humans in New Zealand. N Z Vet J. 2002;50:170–6. 16032266 10.1080/00480169.2002.36306

[5] SAS [computer program]. Version 8.2. Cary (NC): SAS Institute, Inc.; 2000.

[6] Clesceri LS, Greenberg AE, Eaton AD, eds. Standard methods for the examination of water and wastewater. 20th ed. Washington: American Public Health Association; 1998.

[7] Downes FP, Ito K, eds. Compendium of methods for the microbiological examination of foods. 4th ed. Washington: American Public Health Association; 2001.

[8] WHO Collaborating Centre for Reference and Research on Salmonella. Antigenic formulae of the *Salmonella* serovars. Paris: Pasteur Institute; 2001.

[9] Callow BR. A new phage typing scheme for *Salmonella typhimurium.* J Hyg (Lond). 1959;57:346–59. 10.1017/S0022172400020209 13807011 PMC2218121

[10] Barrett TJ, Lior H, Green JH, Khakhria R, Wells JG, Bell BP, Laboratory investigation of a multistate food-borne outbreak of *Escherichia coli* O157:H7 by using pulsed-field gel electrophoresis and phage typing. J Clin Microbiol. 1994;32:3013–7. 7883892 10.1128/jcm.32.12.3013-3017.1994 PMC264217

[11] Tizard IR, Fish NA, Harmeson J. Free flying sparrows as carriers of salmonellosis. Can Vet J. 1979;20:143–4. 385137 PMC1789544

[12] McDonald JW, Bell JC. Salmonellosis in horses and wild birds. Vet Rec. 1980;107:46–7. 10.1136/vr.107.2.46 7434546

[13] Penfold JB, Amery HC, Peet PJ. Gastroenteritis associated with wild birds in a hospital kitchen. BMJ. 1979;2:802. 391319 PMC1596389

[14] Kapperud G, Lassen J, Hasseltvedt V. *Salmonella* infections in Norway: descriptive epidemiology and a case-control study. Epidemiol Infect. 1998;121:569–77. 10.1017/S0950268898001551 10030706 PMC2809564

[15] Hedberg CW, White KE, Johnson JA, Edmonson LM, Soler JT, Korlath JA, An outbreak of *Salmonella enteritidis* infection at a fast-food restaurant: implications for foodhandler-associated transmission. J Infect Dis. 1991;164:1135–40. 1955712 10.1093/infdis/164.6.1135

